# An efficient protocol for isolation of inhibitor-free nucleic acids even from recalcitrant plants

**DOI:** 10.1007/s13205-016-0375-0

**Published:** 2016-02-13

**Authors:** Mohammad Hossein Rezadoost, Mojtaba Kordrostami, Hassan Hassani Kumleh

**Affiliations:** Department of Biotechnology, Faculty of Agricultural Sciences, University of Guilan, P.O. Box: 41635-1314, Rasht, Iran

**Keywords:** *Betula pendula*, DNA isolation, Polysaccharides, Polyphenol, RNA isolation, *Vitis vinifera*

## Abstract

For fast and easy isolation of inhibitor-free genomic DNA even from the toughest plant leaf samples, including those high in polyphenols and polysaccharides, a protocol has been developed. To prevent the solubility of polysaccharides in the DNA extract, high salt concentration (1.4 M) was used in the extraction buffer. Polyvinylpyrrolidone (PVP) was used for the removal of polyphenols as polymerase chain reaction (PCR) inhibitors. Proteins like various enzymes were degraded by proteinase K and removed by centrifugation from plant extracts during the isolation process resulting in pure DNA and RNA ready to use in downstream applications including PCR, quantitative polymerase chain reaction (qPCR), ligation, restriction and sequencing. This protocol yielded a high molecular weight DNA and RNA isolated from leaves and roots of recalcitrant plants which was free from contamination and color. The average yields of total RNA from roots and shoot of Betula and Grape ranged from 285 to 364 ng/µl with A260/A280 between 1.9 and 2.08. The RNA isolated with this protocol was verified to be suitable for PCR, quantitative real-time PCR, semi-quantitative reverse transcription polymerase chain reaction, cDNA synthesis and expression analysis. This protocol shown here is reproducible and can be used for a broad spectrum of plant species which have polyphenols and polysaccharide compounds.

## Introduction

The isolation of high-quality DNA and RNA is important in any molecular biology work because contaminants such as proteins, polyphenols and polysaccharides may interfere with enzymes such as restriction enzymes (in blotting techniques) and *Taq* polymerase [in polymerase chain reaction (PCR)] (Angeles et al. 2005). Isolation of high-quality nucleic acids from plant tissues rich in polysaccharides and polyphenols is often a difficult task. The presence of these substances can affect the quality and/or quantity of the nucleic acids isolated (Heidari et al. 2011). Polysaccharide contamination is a common problem in higher plant DNA and RNA extraction. DNA samples are often contaminated with polysaccharides, polyphenols, which are almost insolvable in water or Tris–EDTA (TE) buffer and are difficult to separate from DNA and RNA. These contaminants are readily identified as they impart a sticky gelatinous brown color to the DNA isolated and interfere with polymerases, ligases and restriction enzymes (Ogunkanmi et al. 2008).

Plant metabolites such as polysaccharides have a similar structure of nucleic acids and are not efficiently removed by most homebrew DNA and RNA isolation methods. Furthermore, the structural similarity allows contaminating polysaccharides in DNA and RNA preparations to interfere with the action of enzymes such as DNA polymerase and reverse transcriptase. Natural substances contained in plant tissues (shoots and roots), such as polysaccharides, inhibit polymerase chain reaction (PCR) to differing degrees. In particular, acidic polysaccharides are extremely strong PCR inhibitors. In this study, to prevent the solubility of polysaccharides in the DNA and RNA extract, high salt concentration (1.4 M) in the extraction buffer was used. In addition, polyvinylpyrrolidone (PVP) was included as an optional step for samples high in polyphenolic compounds, such as, Betula and grape leaves. This compound breaks the bond between DNA and RNA and phenolics, preventing loss of DNA and increasing DNA yield.

Although many protocols have been published for the isolation of total RNA from different plant tissues, the majority are not completely satisfying as they may be time consuming (Yin et al. 2011; Porto et al. 2010), technically complex (Carra et al. 2007; Ren et al. 2008), require ultracentrifugation steps (Carra et al. 2007) and are specific to a particular plant species (Ma and Yang 2011).

To our knowledge, this is the first report of a highly efficient method to extract DNA and RNA from roots and shoots of the recalcitrant plants.

## Materials and methods

### Plant materials

The fresh leaves and roots were collected from different plant species like Betula (*Betula pendula*), and grape (*Vitis vinifera*) in Iran and were taken for laboratory. For each sample, three parcels were made and reserved in −70 refrigerators until DNA extraction. *Betula* *pendula* and *Vitis vinifera* are recalcitrant species with high levels of polysaccharides, polyphenols and other sticky substances. DNA and RNA extraction from *Betula* has been always hard and phenolic compounds make DNA purity very low.

### Buffers

Buffer 1: 200 mM Tris–HCl, 1.4 M NaCl, 0.5 % (v/v) Triton X-100, 3 % (w/v) CTAB, 0.1 % (w/v) PVP (add to buffer only before use).

Buffer 2: 50 mM Tris–HCl, 2 M guanidinethiocyanate, 0.2 % (v/v) mercaptoethanol (add to buffer only before use), 0.2 mg/ml Proteinase K (add to buffer only before use).

### Reagents

2 M Sodium acetate, 2 M LiCl, 4 M NaCl, chloroform–isoamylalcohol (24:1, v/v), isopropanol, 75 % (v/v) ethanol (EtOH).

### DNA isolation

1. Scrap 50 mg of leaf tissue in a 2-ml tube.
2. Add 400 µl buffer 1 and 0.1 % (w/v) PVP, vortex for 20 s and transfer the tube to the heat sink at 60 °C for 30 min.
3. Add 400 µl chloroform–isoamylalcohol (24:1, v/v) and shake severely for 2 min.
4. Centrifuge the tube for 15 min at 10,000 rpm.
5. Transfer 300 µl of supernatant to a fresh 2-ml sterilized centrifuge tube and add 1/2 volume Buffer 2 and transfer the tube to heat sink at 40 °C for 15 min.
6. Add 1/2 of total volume 4 M NaCl, shake and place the tube on ice for 5 min.
7. Add 2 volume cold isopropanol and place at room temperature for 2 min.
8. Centrifuge at 8000 rpm for 15 min (in this stage, the pellet should be seen).
9. Discard the supernatant.
10. Wash the pellet with 75 % (v/v) ethanol (add ethanol gently and keep for 2 min at room temperature, do not spin, be careful that the pellets do not spill out, then centrifuge at 8000 rpm for 2 min).
11. Dry the pellet and dissolve in the 100 µL TE buffer.
12. Transfer the tube containing DNA to heat sink at 70 °C for 10 min.

### RNA isolation

1. Scrap 50 mg of leaf tissue in a 2-ml tube.
2. Add 400 µl buffer 1 and 0.1 % (w/v) PVP, vortex for 20 s and transfer the tube to the heat sink at 60 °C for 30 min.
3. Add 400 µl chloroform–isoamylalchol (24:1, v/v).
4. Add 0.1 volume 2 M sodium acetate (2 M sodium acetate preparation for RNA extraction: add 16.42 g sodium acetate (anhydrous) to 40 ml water and 35 ml glacial acetic acid. Adjust to a pH of 4 with glacial acetic acid and bring to a final volume of 100 ml with DEPC-treated water).
5. Shake severely for 2 min and place the tube on ice for 15 min.
6. Centrifuge at 10,000 rpm at 4 °C for 20 min.
7. Transfer 300 µl of supernatant to a fresh 2-ml sterilized centrifuge tube and add 1/2 volume Buffer 2 and transfer the tube to heat sink at 40 °C for 15 min.
8. Add 1/2 of the total volume 2 M LiCl and keep for 10 min on ice.
9. Add 2 volume isopropanol and store for 1 h at −20 °C.
10. Centrifuge at 12,000 rpm at 4 °C for 20 min (in this stage the pellet should be seen).
11. Wash the pellet with 75 % ethanol (add ethanol gently and keep for 2 min at room temperature, do not spin, be careful that the pellets do not spill out then centrifuge at 8000 rpm for 2 min).
12. Dry the pellet and dissolve in 100 µl DEPC-treated water.
13. Transfer the tube containing RNA to heat sink at 70 °C for 10 min.

**Fig. 1:**
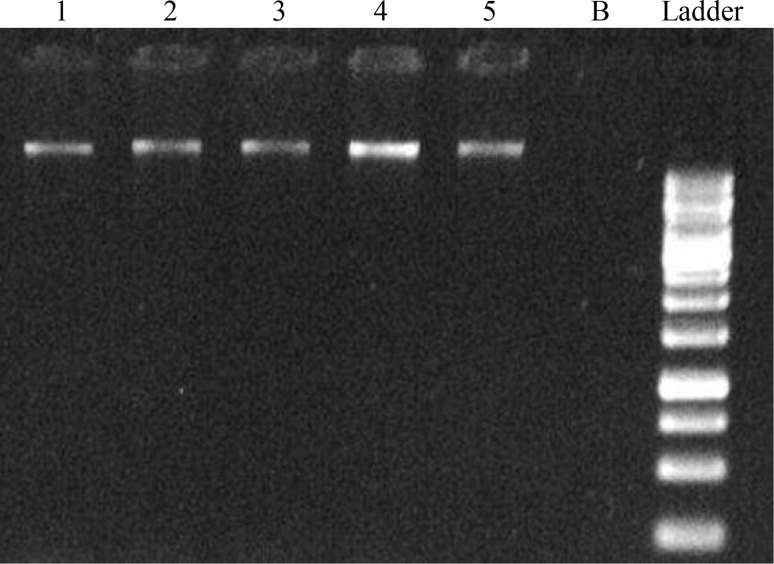
Gel electrophoresis of extracted DNA (B: *Betula pendula*, V: *Vitis vinifera*). *1*–*3*: *Betula* leaf and root DNA, *4*: *Vitis* leaf, *5*: *Vitis* root DNA

Quantification and qualification of isolated DNA and RNA were assayed by spectrophotometry and 1D electrophoresis gel analysis by Total Lab (TL 120). Extracted DNA was first checked on a 0.8 % agarose gel. After electrophoresis, it was stained with ethidium bromide and viewed under an ultraviolet transilluminator for quality and yield assessments (Fig. 1). Because of the high content of secondary metabolites in Betula, AFLP (that its first step is digestion) and RAPD analyses (based on polymerase chain reaction) also were designed to ensure DNA integrity.

The concentration of RNA was determined by measuring the absorbance at 260 nm (A260) in a spectrophotometer using quartz cuvettes. To ensure significance, readings should be between 0.15 and 1.0. An absorbance of 1 unit at 260 nm corresponds to 40 μg of RNA per ml. This relation is valid only for measurements made at neutral pH. Therefore, if it is necessary to dilute the RNA sample, this should be done in a low-salt buffer with neutral pH (e.g., 10 mM Tris–Cl, pH 7.0). When measuring RNA samples, cuvettes were checked to be RNase free, especially if the RNA is to be recovered after spectrophotometry. This can be accomplished by washing cuvettes with 0.1 M NaOH, 1 mM EDTA followed by washing with RNase-free water.

### AFLP analysis

**Fig. 2:**
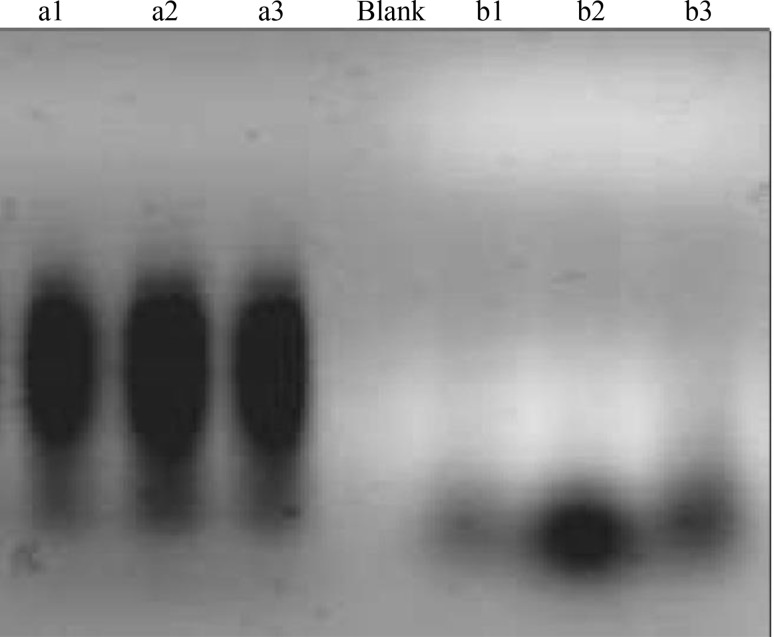
Two main steps of AFLP analysis in this study. *Left* (*a1*–*a3*): pre-selective step in Betula and Grape genotypes made a strong smear on agarose gel. *Right* (*b1*–*b3*): *Eco*RI and *Mse*I double digest made light smear

AFLP analysis was performed as described by Vos et al. (1995). At the first step: genomic DNA of Betula and grape populations was digested with two restriction enzymes, *Eco*RI and *Mse*I, and the combination of genomic DNA and restriction enzymes were incubated for 12 h at 30 °C. Figure 2 shows the restriction enzyme (three right lanes) and pre-selective (three left lanes) steps. Long smears show the successfulness.

**Fig. 3:**
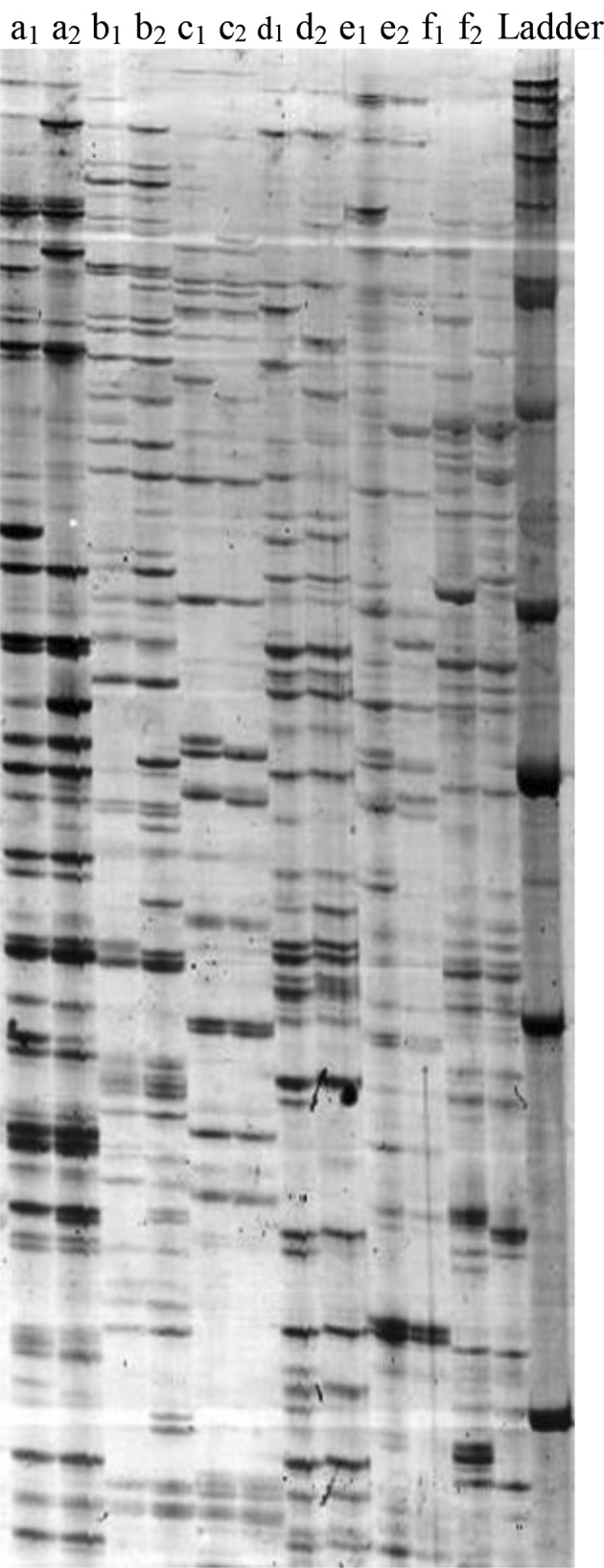
Two *Betula* and *Vitis* genotypes which were amplified on PAGE successfully. *a*_1_, *a*_*2*_; *b*_*1*_, *b*_*2*_; *c*_*1*_, *c*_*2*_: two *Betula* genotypes which were amplified by E32-M61, E36-M42 and E39-M39, respectively. *d*_*1*_, *d*_*2*_; *e*_*1*_, *e*_*2*_; *f*_*1*_, *f*_*2*_: two *Vitis* genotypes which were amplified by E32-M61, E36-M42 and E39-M39, respectively

In the second step: the two stranded adaptors were ligated to the restricted fragments. After that the pre-selective amplification, a subset of all the fragments was amplified, using primers that are complementary to the linker sequences. In the last step, the number of fragments was further reduced by a second round of PCR (selective amplification), in which the PCR primers had an additional three selective bases (Meudt and Clarke 2007). The PCR products were separated on denaturing 6 % polyacrylamide gels and the bands were revealed using the silver staining protocol (Panaud et al. 1996) (Fig. 3). The primers used for AFLP analysis are listed in Table 1.

**Table 1:** AFLP primers used for this study

Primer	Sequence
*Mse*I	5′-GATGAGTCCTGAGTAA-3′
*Eco*RI	5′-GTAGACTGCGTACCAATTC-3′
*Mse*I adaptor	5′-GACGATGAGTCCTGAG-3′
*Mse*I adaptor	3′-TACTCAGGACTCAT-5′
*Eco*RI adaptor	5′-CTCGTAGACTGCGTACC-3′
*Eco*RI adaptor	3′-CATCTGACGCATGGTTAA-5′
E32-M61	Eco-**AAC** + Mse**CTG**
E36-M42	Eco-**ACC** + Mse**AGT**
E39-M39	Eco-**AGA** + Mse**AGA**

### RAPD-PCR

**Fig. 4:**
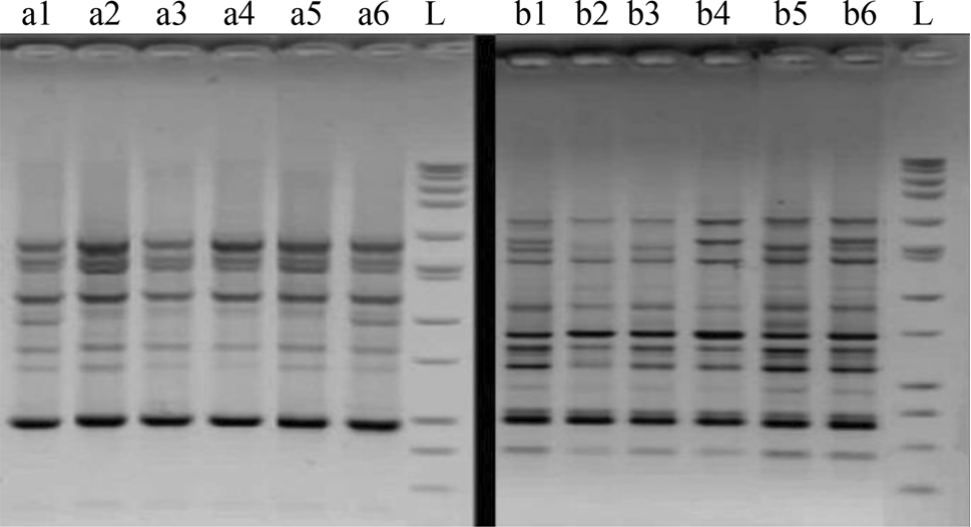
Amplified RAPD patterns of genomic DNA of *Betula* (*a1*–*a6*) and *Vitis* (*b1*–*b6*) genotypes which were amplified with OB10 primer

Six decanucleotides of arbitrary sequence were tested for PCR amplification to assess the genetic variability of the samples: BB13, OA12, OA4, OB10, OB20 and OC4 (Table 2). Amplification reaction was performed according to the method described by Saker et al. (2005) with slight modification, which contains a template (1.5 μl), primer (1 μl), enzyme master mix (12.5 μl) and Milli Q water (10 μl). The amplification was carried out in a DNA thermal cycler with PCR profile: pre denaturation at 94 °C for 5 min, 36 cycles at 94 °C for 30 s, 36 °C for 30 s, and 72 °C for 1 min, with a final extension at 72 °C for 5 min, finally amplified product was held at 4 °C. The 15 μl of amplified products were resolved in 2 % agarose gel with ethidium bromide. The electrophoresis gel was documented under UV light (Fig. 4).

**Table 2:** RAPD primers used for this study

Primer	Sequence
BB13	5′-TTCCCCCGCT-3′
OA12	5′-TCGGCGATAG-3′
OA4	5′-AATCGGGCTG-3′
OB10	5′-CTGCTGGGAC-3′
OB20	5′-GGACCCTTAC-3′
OC4	5′-CCGCATCTAC-3′

## Result and discussion

### DNA quantification

In this study, we used several protocols of DNA isolation reported by Cheng et al. (2003), Xu et al. (2004) and Hameed et al. (2004); but a high yield and quality of DNA was only obtained with our modified method. The DNA samples prepared by this protocol were of high purity with low polysaccharide and protein contamination, which was indicated by the A260/A230 and A260/A280 ratios (Sambrook and Russell 2001), which ranged from 1.85 to 2.13 and 1.79 to 1.90, respectively. Spectrophotometry data showed average 292 ng/µl with 260/280 ratio 1.79 for *Betula pendula* and average 324 ng/µl with 260/280 ratio 1.9 for *Vitis vinifera*. Data analyzed by Total Lab (TL 120) indicated that DNA quantities on agarose gel were average 1517.69 ng for *Betula pendula* and 1588.75 ng for *Vitis vinifera*. However, the average genomic DNA yields from other methods ranged from 780 to 1100 ng for young leaves and roots (Table 3).

**Table 3:** Yield and purity of genomic DNA prepared by the new protocol and other methods evaluated by UV light absorption spectra and ratios of A260/A230 and A260/A280

Method	Plant	Tissue	Absorbency ratio		DNA yield (ng/µl)
			A260/230	A260/280	
Our new protocol	Betula	Leaf	1.72	1.79	292
		Root	1.68	1.71	254
	Grape	Leaf	1.76	1.80	324
		Root	1.70	1.78	287
Cheng et al. (2003)	Betula	Leaf	1.28	1.23	132
		Root	1.41	1.44	110
	Grape	Leaf	1.23	1.23	113
		Root	1.52	1.5	98
Xu et al. (2004)	Betula	Leaf	1.12	1.16	87
		Root	1.27	1.25	91
	Grape	Leaf	1.34	1.33	77
		Root	1.44	1.41	101
Hameed et al. (2004)	Betula	Leaf	1.08	1.12	64
		Root	1.34	1.38	63
	Grape	Leaf	1.49	1.44	77
		Root	1.47	1.52	56

### AFLP and RAPD fingerprinting

Good quality starting DNA is one of the most important prerequisites for successful AFLP analysis. For that reason the AFLP profile was tested. The new protocol showed sharp bands and presented polymorphic and scorable bands (Fig. 4). The RAPD procedure had the same situation. According to Fig. 4 it can be concluded that the new protocol presented pure DNA and sharp band than can be scored easily.

### RNA quantification

**Fig. 5:**
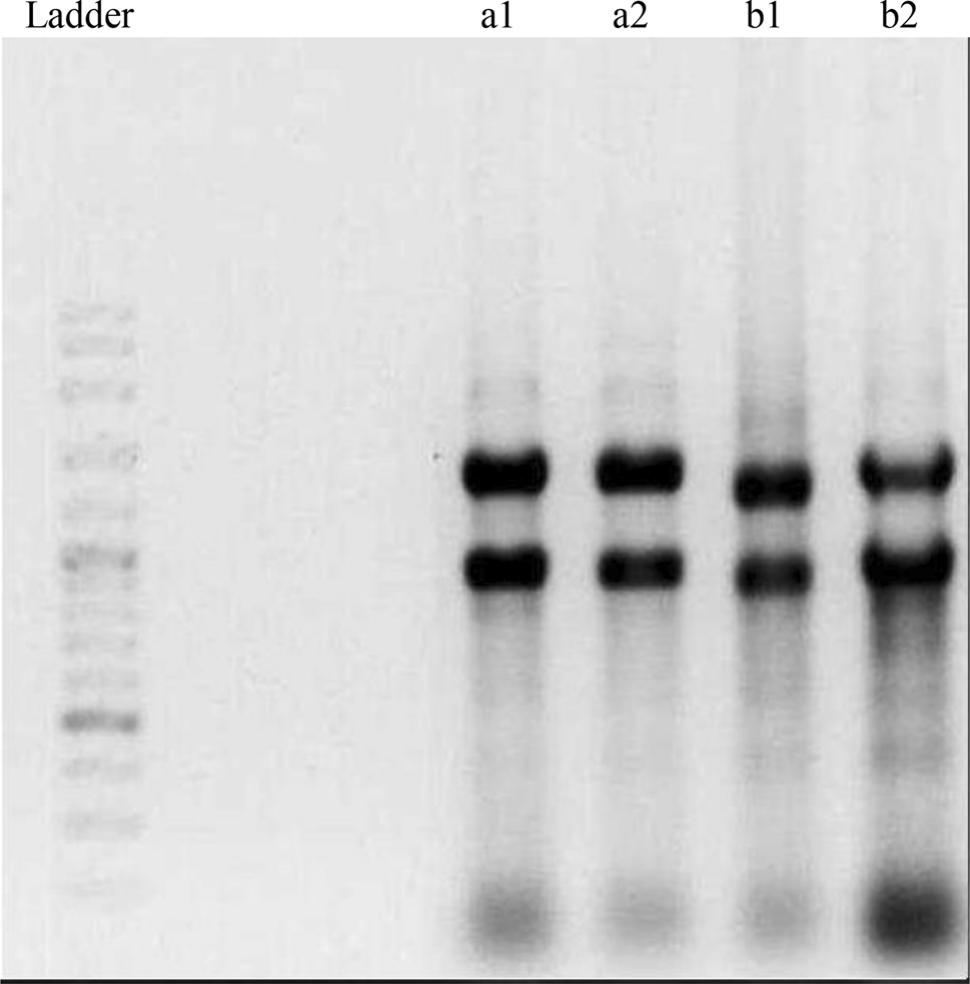
Formaldehyde agarose gel of total RNA isolated from Betula and grape. 1 μl RNA was loaded per lane. *a1* and *a2*: *Betula* leaf and root, *b1* and *b2*: *Vitis* leaf and root, respectively

The RNA samples prepared by this method demonstrated the intact, sharp 28S and 18S ribosomal RNA (rRNA) bands and the lack of RNA degradation on agarose gels, indicating high quality of obtaining total RNA (Fig. 5), whereas other procedures exhibited a failure production of a high-quality RNA due to the serious degradation of the RNA as indicated by the degradation of 28S and 18S rRNA as well as a smear of smaller sized RNAs.

In addition, RNA quality was measured by means of spectrophotometric ratios that relate differences in absorption spectra maxima of pure nucleic acids (Amax = 260 nm), proteins (Amax = 280 nm) and polysaccharides (Amax = 230 nm). In contrast to other methods (Asif et al. 2000; Iandolino et al. 2004; Reid et al. 2006; RNX-PLUS kit, CinnaGen, Iran and Trizol reagent) the A260/A230 ratio for all RNA samples prepared by our protocol was higher than 1.82. This indicated that the RNA samples were of high purity and without polyphenol and polysaccharide contamination. Also, the A260/A280 ratios ranged from 1.87 to 2.01, indicating a low protein contamination (Table 4). On the other hand, the average A260/A230 of RNA prepared by other protocols ranged from 1.01 to 1.44, which indicated that the samples contained polyphenol and polysaccharide contamination. Nevertheless, the average RNA yields from other protocols were far less and ranged from 54 to 112 ng/µl (Table 4).

**Table 4:** Yield and purity of total RNA prepared by the new protocol and other methods evaluated by UV light absorption spectra and ratios of A260/A230 and A260/A280

Method	Plant	Tissue	Absorbancy ratio		RNA yield (ng/µl)
			A260/230	A260/280	
Our new protocol	Betula	Leaf	2.01	1.99	292
		Root	1.98	1.87	285
	Grape	Leaf	2.04	2.01	364
		Root	1.96	1.97	356
Asif et al. (2000)	Betula	Leaf	1.17	1.23	85
		Root	1.21	1.45	97
	Grape	Leaf	1.12	1.31	101
		Root	1.08	1.04	65
Iandolino et al. (2004)	Betula	Leaf	1.01	1.40	112
		Root	1.22	1.22	62
	Grape	Leaf	1.17	1.31	54
		Root	1.22	1.14	69
Reid et al. (2006)	Betula	Leaf	1.19	1.01	79
		Root	1.24	1.22	68
	Grape	Leaf	1.44	1.28	108
		Root	1.38	1.58	103
RNX-PLUS kit	Betula	Leaf	1.08	1.12	54
		Root	1.34	1.31	48
	Grape	Leaf	1.25	1.23	62
		Root	1.14	1.44	71
Trizol reagent	Betula	Leaf	1.32	1.42	110
		Root	1.34	1.13	108
	Grape	Leaf	1.35	1.38	98
		Root	1.48	1.32	112

For as long as scientists have used the polymerase chain reaction (PCR), PCR inhibitors have been obstacles to success. All who use PCR are likely to be impacted by inhibitors at some time, but the wide range of forensic sample types and variety of sampling conditions encountered make forensic scientists particularly vulnerable (Bessetti 2007). It is hard to determine all of the causes of inhibition on the PCR. The PCR process can be affected by compounds that interfere with the interaction between DNA and *Taq* polymerase, and thus inhibit the reaction (Wilson 1997). Many inhibitors are removed during the extraction process through ethanol precipitation or centrifuge process. However, some inhibitors co-elute with the DNA, which may lead to PCR inhibition. A number of inhibitors are contained in the samples themselves, while others can be introduced by the substrate or the analysis process (Bourke et al. 1999). The presence of inhibitors can result in loss of data or results that could be mistaken for degradation. Not all of the factors affecting inhibition are known, and most of the methods used to overcome inhibition are specific to the inhibiting compound.

In this research a protocol has been developed for easy isolation of inhibitor-free genomic DNA from even the toughest plant leaf samples, including those high in polyphenols and polysaccharides. To prevent the solubilization of polysaccharides in the DNA extract, high salt concentration (1.4 M) in the extraction buffer was used in the precipitated DNA. The presence of polysaccharides in extracted plant DNA is a common concern for plant molecular biologists; however, the data presented here show that in many cases this can be averted with the use of increased salt concentrations in extraction buffer (Page 2000). According to Fang et al. (1992) results, high-salt buffer (1.5–2.0 M NaCl) can prove effective isolation of genomic DNA from muskmelon, cucumber, potato, soybean, and geranium. At this level, the polysaccharides remained in the solution and were discarded with the ethanol supernatant, decreasing the levels of polysaccharide. The diatomite procedure described here is quick, simple and most reliable enabling the processing of a large number of samples with ease.

PVP was used for the removal of polyphenols that are known as PCR inhibitors and proteins like various enzymes were degraded by proteinase K and were removed by centrifugation from plant extracts during the isolation process resulting in pure DNA and RNA that are ready to be used in downstream applications including PCR, quantitative PCR, real-time PCR and sequencing. Polyphenolics occur at different concentrations in the leaves, bark and fruit of higher plants. An important characteristic of many polyphenolics is their propensity to form complexes with nucleic acids. Hence, a variety of protocols have been developed to avoid inhibition of molecular biological reactions (Koonjul et al. 1999). In this research, we have included PVP in the extraction buffer, alleviating the inhibition of *Taq* DNA polymerase associated with unknown components, polyphenols, present in several crude DNA preparations and thus increasing the utility of our simple method. It is exceptionally good at absorbing polyphenols during DNA purification. Polyphenols are common in many plant tissues and can deactivate proteins if not removed and, therefore, inhibit many downstream reactions like PCR.

The first step in DNA isolation is to break open the cell and release the cytoplasmic contents, which includes the nucleus, in which we find DNA. Proteinase K is a protease which is used to digest the cell surface proteins. When cell surface proteins are digested, the integrity of the cell membrane is compromised leading to cell lysis. Most protocols for the extraction of DNA from fresh tissue or cultured cells require tissue to be incubated with proteinase K for 12–24 h. An incubation time of 18 h for the proteinase K extraction technique was a very efficient procedure, capable of extracting high molecular weight DNA (more than 20 kilobases) from as little as one frozen section of the fresh tonsil (Jackson et al. 1990). In this protocol, the tissues were incubated with proteinase K for 15 min.

RNA extraction relies on good laboratory technique and RNase-free technique. RNAse is heat stable and refolds following heat denaturation. They are difficult to inactivate as they do not require cofactors. The most common isolation methods can be divided into two classes: utilization of 4 M guanidinium thiocyanate and utilization of phenol and SDS (Chee Tan and Chin Yiap 2009). Guanidinium thiocyanate (GITC) is a chemical compound used as a general protein denaturant, being a chaotropic agent, although it is most commonly used in the extraction of DNA and RNA. Guanidinium thiocyanate is also used to lyse cells, where its function, in addition to its lysing action, is to prevent the activity of RNase enzymes and DNase enzymes by denaturing them. These enzymes would otherwise damage the extract (Shimomura et al. 1978). At the end of experiments, it was found DNA and RNA isolation from these recalcitrant plants are very difficult even using best kits. However, this protocol shown is reproducible and can be used for a broad spectrum of plant species which have polyphenols and polysaccharide compounds.
